# Partial protective effect of *CCR5-Delta 32 *heterozygosity in a cohort of heterosexual Italian HIV-1 exposed uninfected individuals

**DOI:** 10.1186/1742-6405-3-22

**Published:** 2006-09-25

**Authors:** Enrico M Trecarichi, Mario Tumbarello, Katleen de Gaetano Donati, Enrica Tamburrini, Roberto Cauda, Christina Brahe, Francesco D Tiziano

**Affiliations:** 1 Department of Infectious Diseases, Catholic University, Rome, Italy; 2 Department of Medical Genetics, Catholic University, Rome, Italy

## Abstract

Despite multiple sexual exposure to HIV-1 virus, some individuals remain HIV-1 seronegative (exposed seronegative, ESN). The mechanisms underlying this resistance remain still unclear, although a multifactorial pathogenesis can be hypothesised. Although several genetic factors have been related to HIV-1 resistance, the homozigosity for a mutation in *CCR5 *gene (the 32 bp deletion, i.e. CCR5-Delta32 allele) is presently considered the most relevant one. In the present study we analysed the genotype at *CCR5 *locus of 30 Italian ESN individuals (case group) who referred multiple unprotected heterosexual intercourse with HIV-1 seropositive partner(s), for at least two years. One hundred and twenty HIV-1 infected patients and 120 individuals representative of the general population were included as control groups. Twenty percent of ESN individuals had heterozygous CCR5-Delta 32 genotype, compared to 7.5% of HIV-1 seropositive and 10% of individuals from the general population, respectively. None of the analysed individuals had CCR5-Delta 32 homozygous genotype. Sequence analysis of the entire open reading frame of *CCR5 *was performed in all ESN subjects and no polymorphisms or mutations were identified. Moreover, we determined the distribution of C77G variant in CD45 gene, which has been previously related to HIV-1 infection susceptibility. The frequency of the C77G variant showed no significant difference between ESN subjects and the two control groups.

In conclusion, our data show a significantly higher frequency of CCR5-Delta 32 heterozygous genotype (p = 0.04) among the Italian heterosexual ESN individuals compared to HIV-1 seropositive patients, suggesting a partial protective role of CCR5-Delta 32 heterozygosity in this cohort.

## Findings

Despite multiple sexual exposures to HIV-1 virus, some individuals remain HIV-1 seronegative (exposed seronegative, ESN). Although severe factors have been related to HIV-1 infection resistance, the possible genetic mechanisms underlying this resistance presently remain elusive [1,2]. The most investigated genetic factor associated with HIV-1 infection resistance is the homozygous presence of a 32 bp deletion in *CCR5 *gene (CCR5-Delta 32) [3], i.e. the main co-receptor used by the macrophage (M)-tropic strain of the virus to infect peripheral blood mononuclear cells. The 32 bp deletion leads to the synthesis of a truncated protein which does not allow the proper interaction between HIV-1 and the cell surface, thus preventing virion endocytosis. Only 1% of Caucasian individuals is homozygous for *CCR5-Delta 32 *allele and the frequency of this genotype has been reported to be slightly higher in anti HIV-1 seronegative individuals at high risk of HIV-1-infection [4,5].

Other genetic factors have been reported to be involved in HIV-1 infection susceptibility. It has been suggested that the C77G variant in exon 4 of the *CD45 *gene (CD45-C77G) is more frequent in HIV-1 infected subjects, compared to uninfected individuals [6]. This transversion is responsible for an abnormal splicing of exon 4, leading to the production of a high molecular weight isoform of the protein, normally expressed in the naive T cells but not in the normal activated T cells [7]. Other mutations of this gene have been associated to severe forms of combined immunodeficiency in humans [7].

In the present study we investigated the possible role of CCR5 and CD45 genic variants in the resistance to HIV-1 infection in a cohort of heterosexual Italian ESN individuals.

The Catholic University hospital is a 1,700-bed tertiary care centre with approximately 60,000 patient admissions each year. Patients' provenience is mainly from central and southern Italy, and to a lesser extent, northern Italy. The hospital has a 60-bed unit for the admission of HIV patients and a day-hospital for outpatient care.

The present study includes 30 healthy ESN individuals (cases), partners of HIV-1 infected patients who were in a regular follow-up in our outpatient unit. All ESN individuals referred multiple unprotected heterosexual intercourse for a period of at least two years with their own partners. HIV-1 screening test was performed in these subjects every six months. A randomly selected series of 120 HIV-1 infected patients in follow-up at our outpatient unit and an equal number of individuals from the general population, randomly selected among those who had a genetic test done at the Institute of Medical Genetics of our University, were included as control groups (cases:controls = 1:4). All cases and controls, included in the study upon informed consent, were of Italian origin, thus sharing genetic background.

Genomic DNA was extracted from 5–10 ml of peripheral blood, using salt extraction standard procedures. Hundred ng of DNA were amplified by PCR in standard conditions. For CCR5-Delta 32 allele, a primer pair including the deletion was used (CCR5-D32-F: 5'CTTCATTACACCTGCAGCT3' and CCR5-D32-R: 5'TGAAGATAAGCCTCACAGCC3'); PCR fragments of 196 bp and 164 bp for wt and Delta 32 allele, respectively, were separated on 2% agarose gel. For sequence analysis, wt allele was amplified by two primer pairs (CCR5-F1: 5'ATGGAGGGCAACTAAATACATT3'; CCR5-R1: 5'AGATGACTATCTTTAATGTCTG3'; CCR5-F2: 5'CTCTCATTTTCCATACAGTCAGTATCA3'; CCR5-R2: 5'AAGCCATGTGCACAACTCTGACTG3') and sequenced by using ABI-Prism 310 automatic sequencer (Applera), according to the manufacturer's protocol. For the CD45-C77G allele, a fragment including the mutation was obtained by using primers CD45-F: 5'-GATTGACTACAGCAAAGATGCCC-3' and CD45-R: 5'-CCTCTGTGGTATTAAAAGCACTAGCA-3'; subsequent *Hpa*II digestion of PCR products evidenced the mutated allele after agarose gel electrophoresis. The presence of the C77G variant was confirmed by sequence analysis of PCR products from heterozygous subjects.

Contingency data were analysed by the two tailed χ^2 ^test or Fisher's exact test, and continuous data were analysed by use of the Student's t-test. Significance testing of differences in proportions was done using χ^2 ^test. Ninety-five percent test-based confidence intervals (95% CI) were used to determine the statistical significance of the odds ratios (OR). Two tailed tests of significance at the p < 0.05 level were used to determine statistical significance. The statistical analysis was performed using the software programs Intercooled Stata, version 8, for Windows (Stata Corporation USA).

During the study, about 1700 HIV-1 positive patients were in follow up in our outpatient unit. Thirty of them had HIV-1 seronegative partners despite an history of regular unprotected heterosexual intercourse in the last 2 years.

Seven of 30 ESN subjects (23%) were males and 23 females (77%); the mean age (± SD) was 34 (± 5) years. No statistical significant difference in sex and age distribution was observed between cases and controls.

Six of 30 (20%) ESN individuals were heterozygous for the CCR5-Delta 32 allele compared to 9 of 120 (7.5%) HIV-1 seropositive (p = 0.04) and 12 of 120 (10%) individuals from general population (p = 0.2). None of the cases or of the controls had CCR5-Delta 32 homozygous genotype (table 1).

The frequency of CCR5-Delta 32 allele was 10% in cases, compared to 3.7% (p = 0.04) and 5% (p = 0.2) in HIV-1 seropositive controls and general population, respectively. In all three groups the genotype frequencies observed were in equilibrium, as predicted by the Hardy-Weinberg equation.

**Table 1:** CCR5 and CD45 genotypes in exposed uninfected subjects (ESN), HIV-1 seropositive patients and general population control groups.

Genotype		ESN (%) (n = 30)	HIV-1 seropositive (%) (n = 120)	P^c^	General Population (%) (n = 120)	P^d^
CCR5^a^	wt/wt	24 (80)	111 (92.5)	0.04	108 (90)	ns
	Δ32/wt	6 (20)	9 (7.5)	0.04	12 (10)	ns
	Δ32/Δ32	0 (0)	0 (0)	ns	0 (0)	ns
CD45^b^	Wt/wt	29 (97)	117 (97.5)	ns	117 (97.5)	ns
	C77G/wt	1 (3)	3 (2.5)	ns	3 (2.5)	ns
	C77G/C77G	0 (0)	0 (0)	ns	0 (0)	ns

^a ^WT/WT, homozygous wild type CCR5 genotype; Delta32/Delta32 for homozygous mutant for 32-bp deletion in CCR5 gene and Delta32/wt for heterozygous.

^b ^WT/WT, homozygous wild type CD45 genotype; C77G/C77G for homozygous mutant for the point mutation in exon 4 of CD45 gene and C77G/wt for heterozygous.

^c ^P value calculated comparing ESN individuals vs HIV-1 seropositive control group; p = ns, non significant.

^d ^P value calculated comparing ESN individuals vs General Population control group; p = ns, non significant.

Sequence analysis of the entire open reading frame was performed in all ESN subjects, in order to investigate the presence of further putative polymorphisms or mutations in *CCR5 *gene coding region. The result of this analysis indicated the absence of any variation.

CD45-C77G genotype analysis revealed that only one out of 30 ESN individuals (3%) was heterozygous for this variant. In both control groups, 3 out of 120 (7.5%) subjects had heterozygous genotype (p = 0.8). None of the individuals among cases or controls was homozygous for CD45-C77G. The allelic frequencies of CD45-C77G were 1.7% in ESN subjects and 1.3% in both control groups. None of the individuals analysed had double heterozygous genotype at *CCR5 *and *CD45 *loci.

There was no significant difference in the frequency of CD45-C77G polymorphism between ESN and the control groups, suggesting that in our cohort this variant is not involved in HIV-1 infection resistance or susceptibility. No homozygous subject was found for CCR5-Delta 32 mutation in all three groups analysed, which is not surprising considering the overall low frequency of this genotype (table 1). The frequency of the CCR5-Delta 32 heterozygotes observed in our sample of the general population (10%) is very similar to the mean frequency (9.1%) reported in other European studies [8,9]. In ESN individuals we found a statistically significant higher frequency of CCR5-Delta 32 heterozygous genotype (20%), compared to the control group of HIV-1 seropositive individuals (7.5%).

It has been previously reported [3] that homozygous individuals for CCR5-Delta 32 are protected against HIV-1 infection. At the heterozygous state, this mutation has been associated with a slower progression of the disease in HIV-1 infected individuals [2]. However, whether this mutation might have a protective role also in heterozygous ESN individuals, presently remains controversial. No significant difference in the distribution of CCR5-Delta 32 heterozygous genotype between ESN and HIV-1 seropositive individuals has been previously reported in four studies [10-13].

Interestingly, a partial protection against HIV-1 infection has been observed in three different studies [14-16]. In particular, Hoffman et al [14] described a significantly higher frequency of CCR5-Delta 32 heterozygous genotype among the uninfected partners of heterosexual discordant couples, but not in homosexual couples. Similar data were reported also by Philpott et al [15] in a large cohort of women from different ethnic and racial background and with different transmission risk factors. In this study [15] the presence of the Delta 32 allele was significantly associated with lower rates of HIV-1 infection among white individuals. Marmor et al [16], analyzing a large sample of individuals, found a protective role of CCR5-Delta 32 allele in uninfected subjects exposed to HIV-1 infection risk through homosexual intercourse.

The discrepancy between these results may be partially explained by the number of individuals included in the different studies and/or by the relative impact of a single genetic factor in the different populations analysed. Further data supporting the hypothesis of a partial protective effect of CCR5-Delta 32 heterozygous genotype have been recently described [17]. In fact, Agrawal et al. [17] reported that Delta 32 protein provides the down-regulation of cell surface expression of the wild type CCR5 and CXCR4 proteins through heterodimerization in CD4+ primary cells infected by a recombinant Adenovirus carrying Δ32 allele. However, additional studies are necessary to better elucidate the role of *CCR5 *gene and of its variants in HIV-1 infection resistance.

To the best of our knowledge, the present is the first study investigating the allelic distribution of the genetic variants CCR5-Delta 32 and CD45-C77G in a cohort of Italian heterosexually HIV-1 exposed and uninfected individuals. Our data suggest a partial protective effect of CCR5-Delta 32 heterozygosis in the Italian ESN cohort population.

## Competing interests

The author(s) declare that they have no competing interests.

## Authors' contributions

EMT carried out the molecular genetic studies.

MT participated in the design of the study and performed the statistical analysis.

ET and KDGD participated in the collection of the blood samples and conception of the study.

RC and CB participated in its design and coordination and helped to draft the manuscript.

FDT conceived of the study, and participated in its design and coordination.

All authors read and approved the final manuscript.
